# Supplementary material for: Cell Response in Free-Packed Granular Systems
Source: ACS Appl Mater Interfaces. 2022 Aug 31;14(36):40469–80. doi: 10.1021/acsami.1c24095 (PMC9773234; doi:10.1021/acsami.1c24095)
Supplement: Supplementary file 1 — am1c24095_si_001.pdf [file am1c24095_si_001.pdf]

Supporting Information

# Cell Response in Free-Packed Granular Systems

*Ana F. Cunha*[1]*, André F. V. Matias*[2,3]*, Cristóvão S. Dias*[2,3]*, Mariana B. Oliveira*[1]**, Nuno A. M. Araújo*[2,3]**, João F. Mano*[1]***

[1]Department of Chemistry, CICECO – Aveiro Institute of Materials, University of Aveiro, 3810-193 Aveiro, Portugal;

[2]Centro de Física Teórica e Computacional, Faculdade de Ciências, Universidade de Lisboa, 1749-016 Lisboa, Portugal;

[3]Departamento de Física, Faculdade de Ciências, Universidade de Lisboa, 1749-016 Lisboa, Portugal

*Corresponding authors

Email: mboliveira@ua.pt, nmaraujo@fc.ul.pt, jmano@ua.pt

## Supplementary Figure

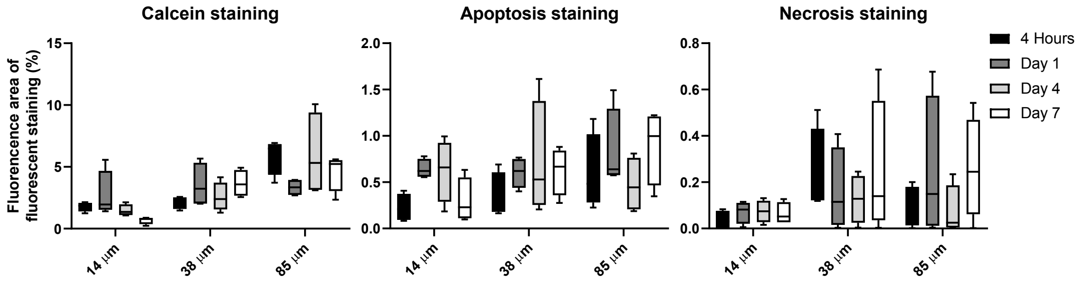


**Figure S1.** Semi-quantification of fluorescent staining of calcein, apoptotic and necrotic markers in hASCs cultured in three different particle beds. Fluorescence microscopy images of live, apoptotic and necrotic stained cells analyzed for the relative fluorescent area of each staining (%); n = 4 independent experiments, 2 images/condition/timepoint.

## Supplementary Videos

**Video S1.** Influence of particle bed conditions on cell spreading: particle beds with different cell-particle aspect ratios in fixed (**a-c**) and free-packing conditions (**d-f**) (MP4).

**Video S2.** Microparticle substrate deposition.
